# High-Fidelity Depth Upsampling Using the Self-Learning Framework †

**DOI:** 10.3390/s19010081

**Published:** 2018-12-27

**Authors:** Inwook Shim, Tae-Hyun Oh, In So Kweon

**Affiliations:** 1 The Ground Autonomy Laboratory, Agency for Defense Development, Daejeon 34186, Korea; iwshim@add.re.kr; 2 Computer Science and Artificial Intelligence Laboratory, MIT, Cambridge, MA 02139, USA; 3 Department of Electrical Engineering, KAIST, Daejeon 34141, Korea; iskweon@kaist.ac.kr

**Keywords:** depth upsampling, depth filtering, LiDAR, self-learning, self-supervised learning

## Abstract

This paper presents a depth upsampling method that produces a high-fidelity dense depth map using a high-resolution RGB image and LiDAR sensor data. Our proposed method explicitly handles depth outliers and computes a depth upsampling with confidence information. Our key idea is the self-learning framework, which automatically learns to estimate the reliability of the upsampled depth map without human-labeled annotation. Thereby, our proposed method can produce a clear and high-fidelity dense depth map that preserves the shape of object structures well, which can be favored by subsequent algorithms for follow-up tasks. We qualitatively and quantitatively evaluate our proposed method by comparing other competing methods on the well-known Middlebury 2014 and KITTIbenchmark datasets. We demonstrate that our method generates accurate depth maps with smaller errors favorable against other methods while preserving a larger number of valid points, as we also show that our approach can be seamlessly applied to improve the quality of depth maps from other depth generation algorithms such as stereo matching and further discuss potential applications and limitations. Compared to previous work, our proposed method has similar depth errors on average, while retaining at least 3% more valid depth points.

## 1. Introduction

In recent research, the advance of the depth sensor has opened a new horizon in the computer vision and robotics field, e.g., scene understanding [1] and object recognition [2], by virtue of the capability of capturing rich 3D information of a scene in real time. Most representative mechanisms of such sensors are categorized into stereo-based range sensors, 3D time-of-flight (3D-ToF), active pattern cameras (e.g., Microsoft Kinect), and light detection and ranging sensors (LiDAR). Among them, the image-based depth sensors, such as stereo vision [3], 3D-ToF [4], and active pattern cameras [5], provide dense depth information, but their performance varies according to the changes of the environmental lighting condition [6], as well as having a limitation of the sensing range [7]. On the other hand, LiDAR sensors [8] have a longer measurable range, are also robust to the effects of environmental lighting, and provide accurate depth sensing. Therefore, they are considered as the most reliable sensors in practical outdoor application scenarios, but the depth data from LiDAR sensors form unorganized sparse point clouds, which often hinder obtaining detailed structural scene understanding due to the scarce resolution compared to any image-based depth sensor.

To overcome the resolution scarcity of LiDAR sensors, applying depth upsampling would be a workaround, which propagates sparse depth points along the guidance of side-information, e.g., a corresponding RGB image of the same scene, and results in a high-quality dense depth map. Given low-resolution depth measurements obtained from low-resolution depth sensors including LiDAR, prior studies [9,10,11] have successfully achieved a high-quality and high-resolution depth map estimation with the additional guidance of a high-resolution RGB image taken from a separate camera sensor under some ideal conditions. In these techniques, a notable condition is that they essentially assume that the depth map and image pair is perfectly-aligned and has negligible alignment errors. This assumption is often not appropriate for robotic visual sensor systems, which commonly have a wide baseline between sensors due to constraints on hardware platform design. It introduces a substantial parallax effect in visual data taken from multiple views, which is a major source of unreliable depth estimation near depth discontinuous regions, and yields flipping points and depth dis-occlusion.

In this paper, we present a new depth upsampling method to obtain a high-resolution and highly reliable depth map guided by an RGB image. This work is the extension of our previous work [12], where a simple heuristic threshold mask was used to filter out unreliable depth after depth propagation by multi-lateral information aggregation. Our key idea of this work is to improve the capability of unreliable depth rejection using a self-learning framework. We train a machine learning model to filter out automatically and adaptively unreliable depth estimates even without exhaustive human annotations. This self-learning framework allows the model to spontaneously adapt to each scene in an online update manner. Furthermore, our high-fidelity rejection framework manages the final dense depth estimation to be tolerant to outlier factors induced by alignment errors of sensors, such as mismeasured depth points (we will denote the mismeasured depth points due to degenerated configurations of the relationship between the object surface and sensors (e.g., extremely slanted surface w.r.t. depth sensor position) as outlier points [13]), flipping points, and dis-occlusion. Our contributions are summarized as follows:

- We propose an RGB image-guided high-quality depth upsampling method robust against specific depth outliers introduced by a depth sensor, e.g., outlier points, flipping points, and dis-occlusion. We design the systematic method consisting of depth outlier handling, RGB image-guided depth upsampling, confidence map estimation, and the self-learning framework to predict high-fidelity depth regions.
- We train our proposed depth map rejection in a self-learning way, which does not require human-annotated supervision labels, but collects training data autonomously.
- Through extensive experiments, we qualitatively and quantitatively validate the effectiveness of our proposed depth upsampling framework. We also demonstrate that our method performs favorably even on the stereo matching scenario.

### Related Work

We review representative robotic sensor systems and depth processing algorithms that exploit depth sensors and RGB cameras.

- Visual sensor system: By virtue of the robustness of the LiDAR sensors, many robotic systems mainly rely on LiDARs along with cameras. For instance, mobility platforms including autonomous vehicles typically use a combined system constituted by the stereo camera and LiDAR sensors [14], and field robots mainly use rotating axial LiDAR and multiple cameras, e.g., Tartan Rescue [15], Atlas [16], and DRC-HUBO+ [17]. On the other hand, instead of expensive LiDARs, robots for indoor activities deploy 3D-ToF or active pattern cameras. These sensors are rarely chosen for outdoor robots because they are often vulnerable to the changes of the environmental lighting condition, e.g., direct sunlight often overwhelms the spectrum range of the light patterns of the active imaging sensors [18]. Thus, improving the LiDAR system by our upsampling method can broaden overall successful application regimes of subsequent algorithms that use the estimated depth information as input.
- Guided depth upsampling: Given a pair of a depth and a high-resolution color image, depth upsampling approaches estimate a high-resolution dense depth map that follows crisp edge information of the color image. Joint bilateral upsampling (JBU) [9] applies a spatially-varying filter to the sparse samples while considering local color affinity and radial distance. Chan et al. [19] accelerated the JBU using a GPU and introduced a depth noise-aware kernel. Dolson et al. [20] presented a flexible high-dimensional filtering method for increased spatial and temporal depth resolution. Park et al. [21] used a least-squares cost function that combines several weighting factors with a non-local structure. Ferstl et al. [10] designed a smoothness term as a second-order total generalized variation and propagated sparse seed points using an anisotropic diffusion tensor obtained from a high-resolution image. In terms of degenerated depth measurements occurred due to dis-occlusion or a distant element of a scene (e.g., sky), all these approaches propagate erroneous observations to a large area if the size of depth hole regions exceeds the algorithmic limit that can be dealt with, e.g., the kernel size limit for the filtering approaches. In our work, we explicitly deal with such erroneous propagation by the initial outlier filtering step and the self-learning-based post-filtering step. This enables us to obtain high-fidelity depth upsampling results.
- Depth outliers’ handling: In practice, sparse seed points used for depth upsampling could often contain outliers. Most typical types of outliers that require separate handling would be flipping points and depth dis-occlusion that occur due to unreliable projection with parallax. Furthermore, there are outlier points, which indicate floating points with intermediate depth values between foreground and background depths occurring around object boundaries. To overcome these issues, Kang et al. [22] detected the flipping points based on the distribution of depth values within a color image segment. Park et al. [21] measured depth variation of the local regions for detecting depth discontinuity and proposed a heuristic approach that identifies flipped depth orders after depth projection. However, their work evaluated the performance of their algorithm on exactly-aligned depth-color image pairs. To a broader extent, ToF depth camera and stereo color camera fusion [23,24] was also introduced. Gandhi et al. [24] investigated specific local regions that had mixed foreground and background depth samples. Georgios et al. [23] grew seeds using a smoothness term that was conditionally defined by an occlusion label obtained from depth discontinuity analysis.

We introduce depth outliers’ handling and depth map upsampling with the self-learning framework. Our depth outliers’ rejection method is not dependent on edge information of the image; therefore, it keeps reliable depth points even in ambiguous image edges. Our filter-based approach can generate a high-fidelity and outlier-free depth map that is not only able to improve the quality of the depth map, but also may increase the success rate of potential subsequent post-task algorithms. Furthermore, our confidence map and self-learning framework can explicitly disregard large holes in a depth map and leave reliable depth regions.

## 2. Materials and Methods

**Figure 1:**
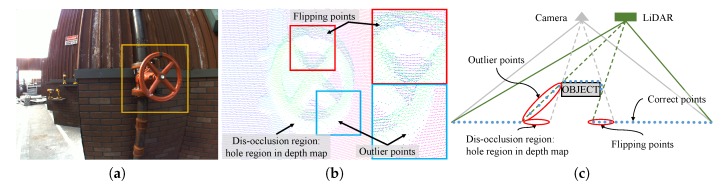
Images showing parallax effects. (**a**) shows a target region; (**b**) depicts registered 3D points to the camera coordinate $P_{C}$, and (**c**) shows why outlier points and flipping points, as well as dis-occlusion problems occur in depth and image alignment.

The first step for depth upsampling is to align a depth and image pair. If we have pre-computed calibration parameters, a depth and image pair can be aligned [12,25]. Figure 1 shows an example of the depth and image alignment with depth errors. Figure 1b shows several erroneous depth points, and (c) depicts the causes of the errors. The outlier points are caused by the measurement noise of the depth sensor and usually occur near the object boundary. The flipping points appear because of the different viewpoints among sensors. The flipping points are regular depth points on the background in the LiDAR coordinate. However, when they are projected on the image, the points are on an object that occludes the background. Therefore, the camera cannot see the corresponding depth points due to occlusion, i.e., no-visibility in the projected viewpoint. The dis-occlusion region is the opposite case of the flipping points. There is no real depth measurement in the dis-occlusion region. These alignment problems can be amplified due to system calibration errors and measurement noise of the depth sensor. For the image-guided depth upsampling algorithms, this unreliability of the alignment severely degrades the performance of depth upsampling, as will be discussed in Section 3.

In this regard, we have to handle these unreliable factors explicitly before performing depth upsampling. We remove outlier points when two adjacent points along a LiDAR scan-line have a considerable distance between them, and we then apply a 2D filter to eliminate isolated sparse points from the image domain. We also remove flipping points by checking the depth information among nearby points. After removing the suspicious points, we run our depth upsampling and generate a confidence map concurrently. Then, we use our proposed self-learning framework to distinguish low-reliability regions including dis-occlusion. We describe this in the following sections.

### 2.1. Early Outlier Rejection

- Outlier points’ rejection: LiDAR sensors could potentially cause depth measurement failure when measuring a light emitted from the sensor that is not adequately reflected due to degenerated surface conditions, such as the extreme edge case of the angle between the light ray and surface normal (edge of an object or a cracked object surface), specific materials with high reflectivity, and so on. These outlier points yield incoherent depth measurements with other close-by correct depth points, i.e., appear as a fractional floating point. Thus, we may eliminate most of these isolated outlier points with a simple 1D or 2D filter. In this paper, we use a simple 1D filter as follows:
  (1) $$P_{f}=\left\{x|max\left(d\left(x_{t}^{l},x_{t-1}^{l}\right),d\left(x_{t}^{l},x_{t+1}^{l}\right)\right)>T_{f}\right\},$$
  where *P_f_* is a set of outlier points, d(·) is the Euclidean distance between two points, and $x_{t}^{l}$ is the $t^{\mathrm{th}}$ point in the $l^{\mathrm{th}}$ scan-line. *T_f_* is a predefined threshold. This filter is applied along every horizontal scan line-by-line. The scan-line stands for a trace of a ray of the LiDAR sensor, the direction of which is congruent with the direction of the rotating axis of the mirror in the LiDAR. In this work, we assume that the horizontal direction of the image is roughly similar to the scan-line direction of the LiDAR, i.e., horizontal scan-line. After that, we use morphological operations in the image to remove isolated sparse points. In some cases, desirable depth points near the object boundaries could be also removed in this process, but we show that it is easily recoverable during the subsequent depth upsampling process.
- Flipping points’ rejection: Most depth upsampling methods assume that a sparse depth and high-resolution color image pair is well aligned, and they do not seriously treat the effect of flipping points, which causes a severe problem in depth upsampling. In this paper, we detect the flipping points by the geometric difference between two sensors and remove them in order to be free from the bad influence of the flipping points.

**Figure 2:**
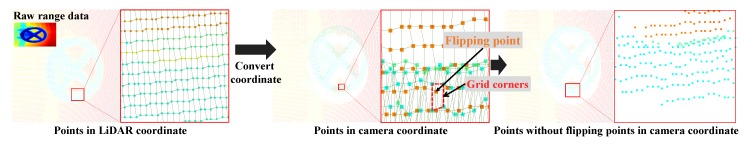
Pipeline for eliminating flipping points using a 4-connected grid map. The outlier and sparse points are removed in advance, which is described in Section 2.1.

Figure 2 shows the process for eliminating flipping points. We first generate a grid map by connecting four nearby points (4-connected grid map) from depth measurements in the LiDAR coordinate. Then, we move the structure of the grid map to the camera coordinate and find points that invade another grid cell, as shown in the center image. Among the points, we reject a point if its depth is more distant than the depth of each corner point of the invaded grid cell. Note that the grid map is for finding the invading points in a grid cell. Because LiDAR provides depth data sequentially, it is easy to construct data indexing for connecting near points at the data-capturing stage and easy to generate a grid map quickly. While we used a four-connected grid map for a simple implementation, one can also use other methods such as Delaunay triangulation [26] to build the grid map.

### 2.2. Depth Map Upsampling and Confidence Map Estimation

In this section, we describe our depth upsampling method and explain how to compute a confidence map of the upsampled depth map.

#### 2.2.1. Depth Map Upsampling

Our depth upsampling algorithm is based on a rolling guidance filter suggested by Zhang et al. [27]. The rolling guidance filter is an iterative joint filter method that can achieve scale-aware local operations; therefore, it is especially useful for removing small-scale structures such as noise while performing edge-preserving upsampling. In our upsampling algorithm, we extend the JBU [9] with an additional depth guidance term to prevent the texture copying problem and use the extended JBU as a joint filter in the rolling guidance filter. Specifically, our upsampling algorithm is formulated as follows:

(2) $$\begin{array}{c} D_{p}^{t+1}=\frac{1}{N_{p}}\sum_{q\in \Omega (p)}\exp\left(G_{p,q}+K_{p,q}+H_{p,q}\right)R_{q},\;\mathrm{where}\;G_{p,q}=-{∥p-q∥}^{2}/2\sigma_{s}^{2},\\ K_{p,q}=-{∥I_{p}-I_{q}∥}^{2}/2\sigma_{i}^{2},\\ H_{p,q}=-{∥D_{p}^{t}-R_{q}∥}^{2}/2\sigma_{d}^{2},\\ N_{p}=\sum_{q\in \Omega (p)}\exp\left(G_{p,q}+K_{p,q}+H_{p,q}\right), \end{array}$$

*I*, *R*, and $D^{t}$ denote a guidance image, an aligned sparse depth map, and an upsampled dense depth map after the $t^{\mathrm{th}}$ iteration, respectively. Here, *p* is a query point, and Ω(p) is a set of neighboring points in the sparse depth map, *R*, within a filter range. $\sigma_{s}$, $\sigma_{i}$, and $\sigma_{d}$ denote the standard deviations to control the influence of the spatial similarity term *G*, the intensity similarity term *K*, and the depth similarity term *H* on the filter weights, and $N_{p}$ is a normalization factor of the weights. For an initial upsampled depth map $D^{0}$, we use the JBU [9] where *H* is set to zero. Equation (2) iteratively estimates a dense depth map, $D^{t}$. The depth-guiding term *H* has an important role, which suppresses error propagation and texture copying problems. Furthermore, it gives vital information in computing the confidence of an estimated depth map.

**Figure 3:**
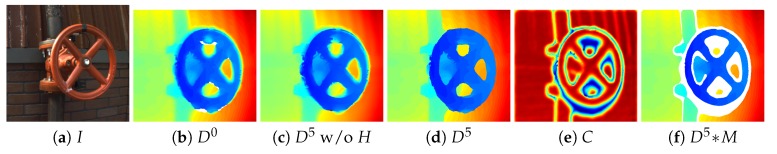
Intermediate results of our depth upsampling method. (**a**) Input image; (**b**) initial depth map $D^{0}$; (**c**) depth upsampling result without the *H* term; (**d**) depth upsampling result after five iterations $D^{5}$; (**e**) confidence map; (**f**) depth upsampling result after masking low reliability regions in white.

Figure 3 shows the intermediate results of our upsampling method. In the figure, our result after five iterations in (d) has sharper and more accurate depth boundaries than the initial upsampled depth map in (b), while the result without the *H* term in (c) has noisy depth boundaries due to overfitting to intensity information.

#### 2.2.2. Confidence Map Estimation

Because of missing depth measurements in the hole regions on the depth map as shown in Figure 1c, the upsampled depth results in those regions are uncertain. It is difficult to calculate the correct depth in this ambiguity by just the upsampling process alone. In this regard, we propose to use a confidence map to deal with this incorrect depth estimate. Our confidence map estimation is closely related to the statistics of measurements, where the small variance of local supporting measurements provides a measure inversely proportional to the confidence of the depth map. Therefore, we define the confidence map *C* as follows:

(3) $$C_{p}=\frac{1}{\max(C)}\sum_{t=0}^{n}\left(\sum_{q\in \Omega (p)}\exp\left(G_{p,q}+H_{p,q}\right)\right),$$

where $C_{p}$ denotes a confidence value on the location *p* and *n* is the number of iterations, and the other notations are equal to Equation (2). This confidence map can be simultaneously computed during the upsampling processing. The notion behind this measure is that a pixel has low confidence if few or unstable depth measurements support the estimated depth. We can mask an estimated depth point out as an unreliable result if its confidence value is lower than a specific threshold value.

Figure 3e shows an example of the confidence map, and (f) is the upsampling result without confidence values lower than 0.35. The confidence mask effectively removes the unreliable depth regions and retains important depth information with clean and sharp depth boundaries. However, a single threshold does not adaptively filter low confidence points. To handle this problem, we adopt the self-learning technique [28] with deep learning architectures [29,30,31]. The details of this issue are presented in Section 2.3.

#### 2.2.3. Parameter Selection

**Figure 4:**
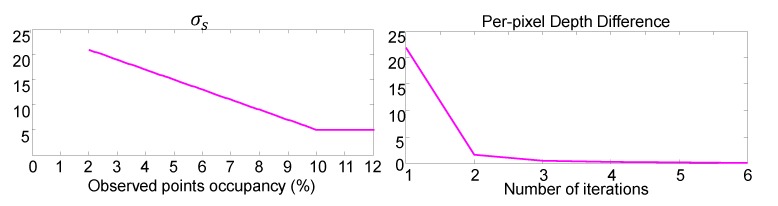
Parameter selection. (**left**) The parameter used for $\sigma_{s}$ according to the occupancy rate of depth points across an entire image, i.e., sparsity; (**right**) the depth variation changes according to the number of iterations, which shows that our rolling guidance scheme converges within only a few iterations.

We have several parameters to use our depth upsampling. First, $\sigma_{s}$ is a spatial smoothness parameter, which is adaptively determined through empirical cross-validation since the proportion of measured depth points to the guidance image pixels may vary according to the sensor systems. The left plot of Figure 4 shows the parameter we used according to the proportion. For example, if the measured points occupy 5% of a guided image area, $\sigma_{s}$ is set to 15. Through experiments, we found that our proposed method requires the depth measurements occupy at least 2% of the image area. Next, $\sigma_{d}$ is a depth similarity parameter to suppress depth measurement noise. $\sigma_{d}$ can be determined based on the error between initial depth $D^{0}$ and depth measurement by a depth sensor. For example, we can determine $\sigma_{d}$ according to the specification of a depth sensor. In the case of UTM 30LX-EW, $\sigma_{d}$ is set to 30 because the maximum repeated accuracy of the LiDAR sensor is less than ±30 mm. We have empirically set the intensity similarity parameter $\sigma_{i}$ to 20 by referring to Zhang et al. [27].

We also need to determine the number of iterations in the rolling guidance scheme. The right plot of Figure 4 shows the average depth variations at each iteration step. The depth map rapidly converges to the final result within 3∼5 iterations.

### 2.3. Self-Learning Framework to Predict High-Fidelity Depth

**Figure 5:**
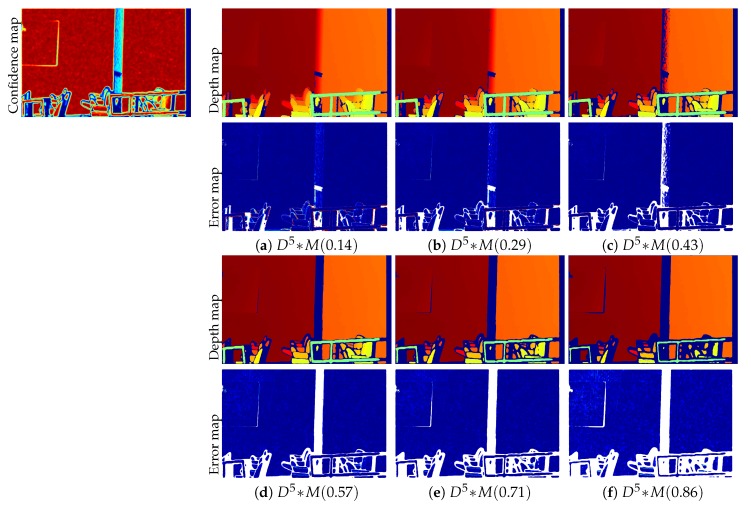
Our upsampled depth maps and their corresponding error maps according to different thresholds *M*. (**a**) M=0.14; (**b**) M=0.029; (**c**) M=0.43; (**d**) M=0.57; (**e**) M=0.71; (**f**) M=0.86. The error maps indicate a relative depth error ranging from 0–3% of the maximum depth. Please see them in the original resolutions to compare all the details.

In our previous work [12], we computed the confidence mask by applying a single threshold value to the entire confidence map (see Figure 3e,f). However, a single threshold value might not be generalizable to diverse environments in order to retain highly reliable depth points. Figure 5 shows our depth upsampling results and their corresponding error maps according to different threshold values. The smaller threshold value keeps a large number of depth points with the relatively large errors especially at the object boundary (a), and the larger threshold value keeps a small number of depth points with relatively small errors (f). This raises a trade-off issue to find desirable thresholds that allow us to obtain a large number of highly reliable depth points with small error.

We transform this threshold decision problem into a pixel-wise binary classification problem with the self-learning framework. The construction of supervised learning systems is time consuming and difficult because a large number of training samples has to be collected and the samples should be manually labeled. To reduce the effort to prepare the training set, semi-supervised learning has been researched, which trains a classifier with both a small number of labeled data and additional unlabeled data [32]. Self-learning is one of the semi-supervised learning approaches. It starts by training a classifier using the small-sized labeled data; then the classifier predicts the unlabeled data. The several positive examples of the estimated data are added to the training set, and the classifier is retrained [33].

In the following sections, we describe the details of our self-learning framework to obtain a high-fidelity depth map. Firstly, we present how to use the confidence map to gather training data autonomously in Section 2.3.1, how to convert this training data into features for learning in Section 2.3.2, and classifiers and online usage of the self-learning framework in Section 2.3.3.

#### 2.3.1. Training Data

**Figure 6:**
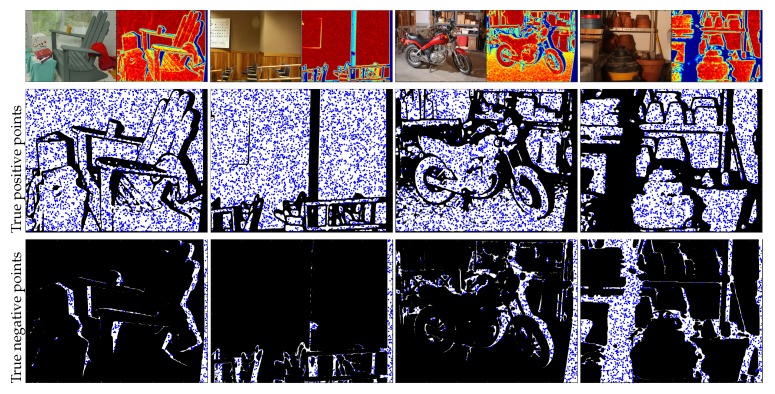
Examples of the extracted training data. The top row shows images and their corresponding confidence maps. The middle and bottom rows show the example of the extracted training points in the high confidence and the low confidence region, respectively. The blue dots depict the positive and negative sets.

Using the reliability information of the confidence map, we split the depth map into two part: true positive and true negative sets. The true positive set is extracted from a very high confidence region in the confidence map. On the contrary, the negative set is extracted from a very low confidence region. Figure 6 shows examples of the true positive and true negative sets.

In our training process, we equally extract the data ratio of the positive and negative sets. In the case of the negative set, the top 20% worst confidence points are used for learning, and we extract the same number of positive samples as follows:

(4) $$D_{pos}=[i_{1},\ldots ,i_{m}],\;D_{neg}=[i_{1},\ldots ,i_{n}],$$

where *m* and *n* are the number of true positive and true negative samples (we set m=n in this work) and *i* denotes the indexes of the extracted samples.

#### 2.3.2. Input Features

We design handcrafted local features considering the color difference, spatial distance, and depth difference between a query point *p* and its neighboring LiDAR measurement points *q*, which are used to compute the upsampling process Equation (2). The seed depth points in *q* are unstructured 3D points’ projection to the 2D image plane (already mentioned in Section 1 and Section 2); thus, those points do not lie on a regular grid structure, but are totally unstructured. In this regard, the off-the-shelf CNN for feature extraction cannot be directly used in our input setup, because CNN requires the input to be a strict regular structured input shape.

Taking this into account, we devise to leverage the statistically-pooled feature (mean and standard deviation with their lower and upper confidences), so that we can capture the distribution characteristics of features, as well as manage to have a fixed structure of the input.

(5) $$F_{p}=\left[f^{r},f^{g},f^{b},f^{d},f^{s}\right],\;\mathrm{where}\;f^{\alpha }={\left[\mu^{\alpha },\sigma^{\alpha },\mu_{l}^{\alpha },\mu_{u}^{\alpha },\sigma_{l}^{\alpha },\sigma_{u}^{\alpha }\right]}^{⊤},\;\alpha \in \left\{r,g,b,d,s\right\},$$

**Figure 7:**
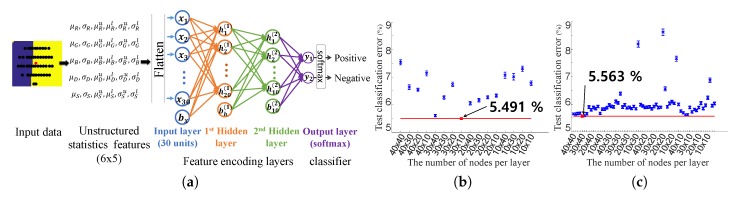
Network model for the adaptive threshold prediction. (**a**) Illustration of the network architecture we use with two hidden layers, which consists of the feature encoding part and the classifier part; (**b**,**c**) ablation study of the mean and variances of the classification errors according to the number of nodes per layer for a network with two hidden layers (**b**) and with three hidden layers (**c**). Each layer of the network is trained with unsupervised pre-training.

$F_{p}$ is a 2D feature matrix of $p^{\mathrm{th}}$ training data. For the sake of notational simplicity, we omit the index of a pixel *p*. The feature matrix includes feature vectors $f^{\alpha }$, which consist of statistics information between the *p* point and its neighboring points, q∈Ω(p), in various feature domains (see Equation (2)). μ and σ are the estimated mean and standard deviation values of the normal distribution fitting given data, which are calculated by the absolute difference between the *p* point and a set of *q* points in each feature domain α. The other parameters in the feature vector are 95% confidence intervals for the parameter estimates on the mean and standard deviation. $\mu_{l}$ and $\mu_{u}$ indicate the lower and upper bounds of the confidence intervals for μ. $\sigma_{l}$ and $\sigma_{u}$ indicate the bound parameters of σ. We exploit color (r,g,b), depth (*d*), and spatial information (*s*) as the feature domain. This feature matrix will be vectorized before feeding into a classifier. Figure 7a depicts the unstructured statistics features.

#### 2.3.3. Classifier with the Online Self-Learning Framework

**Figure 8:**
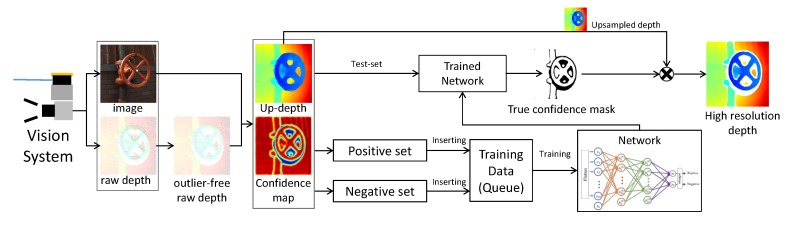
The overall scheme of the self-learning framework. This framework stacks the training data every time when the vision system produces an upsampled depth map and a confidence map. The trained network predicts the true confidence mask for the test-set data.

Figure 8 depicts the whole process of the self-learning framework. Our proposed self-learning framework continuously collects training data ($D_{pos}$ and $D_{neg}$) every sequence. In the very first phase, the self-learning framework is learned by only a small number of training data. Due to the lack of data, the performance of classifiers may be somewhat lower at first. However, the performance will grow according to increasing accumulated training data. We show the performance change according to the number of data accumulation seen in the following.

**Figure 9:**
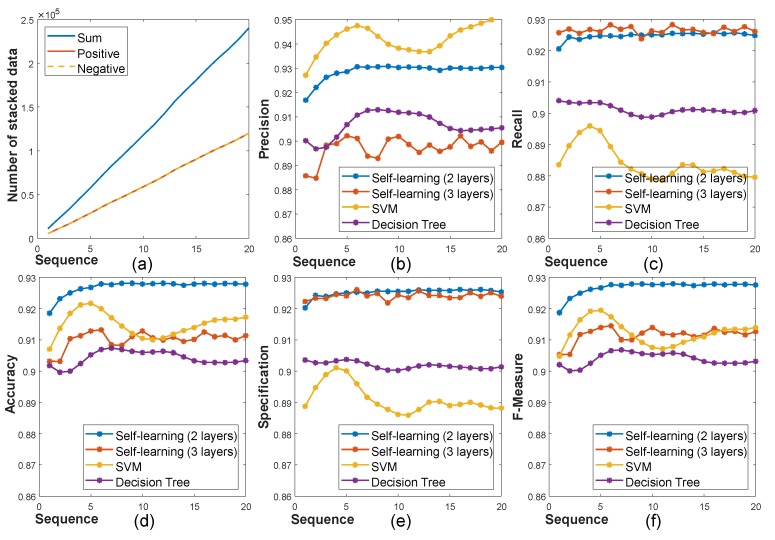
Performance comparison with baseline classifiers according to the number of training data used. (**a**) shows the number of training data, which grows over time, and (**b**–**f**) show the performance of the tested classifiers by the online self-learning framework according to the variations of the number of training data: precision (**b**), recall (**c**), accuracy (**d**), specification (**e**), and F-measure (**f**).

The classifier is utilized to predict the highly reliable depth and filters out low reliability depth by pixel-wise binary classification. We evaluate the performance of some classifiers with the self-learning framework including support vector machine (SVM [34]), decision tree (DT [35]), and fully-connected networks (FC networks [30]). Figure 9 shows the performance comparisons of the classifiers using precision, recall, accuracy, specification, and the F-measure according to the number of data seen in the training set. In the case of precision, the SVM shows better performance than the others, but the recall value is low. This gap between precision and recall means that SVM overestimates depth points to be positive. Among the tested classifier, the FC network with two hidden layers shows the best performance in accuracy, specification, and the F-measure. Figure 9a shows the number of collected data to train. Note that even if the number of stacked training data is increased, the performance of the classifiers is saturated at a specific point. Thus, we manage the training data memory using the queue structure to fix the memory size (∼120,000).

The architecture of the network is depicted in Figure 7a. For simplicity, we omit the nonlinearity activation function, ReLU [36], in the figure, but which is applied to every FC layer. The network contains two FC layers, and the output of the last FC layer is fed to a softmax layer, which provides probability values for binary classification. To determine proper numbers of nodes per layer and the number of layers, we perform the ablation study according to various conditions. Figure 7b,c shows the classification errors according to different numbers of nodes and layers. In these experiments, the network having two hidden layers shows the best performance, which has 30 nodes for the first and 10 nodes for the second hidden layer, and the second best network has three hidden layers, which has 40 nodes for the first, 30 nodes for the second, and 40 nodes for the third layer. We compare the performance of two respective network models with two hidden layers and three hidden layers in Figure 9b–f.

To initialize the FC network, we construct the network by stacking the unsupervised pre-trained autoencoder [31,33] in which the output of each layer is connected to the input of the successive layer. The stacked autoencoder is typically used as a way to pre-train layers in a deep neural network for classification, avoiding the difficulty in the training scheme for such a network as a whole from scratch by performing the greedy layer-wise training method [31,37,38,39]. Each part of the autoencoder is pre-trained in an unsupervised fashion in turn, and then, the overall network is fine-tuned using labeled training data. Through the unsupervised pre-training phase, the network is robust with respect to random initialization, decreasing the probability of finding poor apparent local minima and supporting finding a better solution from training data. According to Erhan et al. [31], the networks with two or three hidden layers with greedy layer-wise pre-training show stable performance for classification tasks.

## 3. Results and Discussion

To validate the performance of our proposed method, we performed experiments on benchmark datasets and compared our method to state-of-the-art methods, such as joint bilateral filter (JBU) [9], nonlocal means (nonlocal) [40], and total generalized variation (TGV) [10]. In Section 3.1, we evaluate and analyze the improved depth accuracy of our proposed method on the Middlebury 2014 dataset [41]. In Section 3.2, we demonstrate that our self-learning framework is seamlessly applicable to the depth obtained from the stereo matching algorithms on the KITTI dataset [42].

In our experiments, we construct the grid map for flipping point rejection using four-connected points. The parameters $\sigma_{s}$ and $\sigma_{i}$ are determined as described in Section 2.2.3. Because $\sigma_{d}$ determines the range of influence of the depth similarity term *H*, $\sigma_{d}$ is determined based on the error between initial depth $D^{0}$ and depth measurement by a depth sensor. In Section 3.1, we used the Middlebury stereo datasets [41] to create data pairs of a high-resolution image and sparse depth data with accurate ground truth, where $\sigma_{d}$ was set to 30 mm under the assumption of additive Gaussian noise. Each of the data in the Middlebury consists of high-resolution stereo images and their corresponding highly accurate dense depth maps estimated by a structured lighting system with calibration parameters. To simulate our data setup (a pair of high-resolution images and a sparse depth), we sampled 2% depth points from the dense depth maps and added additive Gaussian noise (σ=10 mm). Then, the sampled depth was projected onto the other image pair. More details for the dataset generation were described in our previous works [12]. In the case of refining the stereo depth experiment (Section 3.2 KITTI), $\sigma_{d}$ was set to 0.5 m in consideration of stereo matching error. The predefined threshold $T_{f}$, for the outlier point, was set to two times $\sigma_{d}$. For the fixed threshold case, the threshold value of the confidence map was 0.35. We used five iterations for our depth upsampling processing.

For the implementation, we used a 3.6-GHz quad-core CPU and 16 GB RAM. Our CPU-based implementation took about one second to generate a dense depth map of 640 × 480 resolution in pixels with five iterations of joint filtering.

### 3.1. Quantitative Evaluation: Middlebury

**Figure 10:**
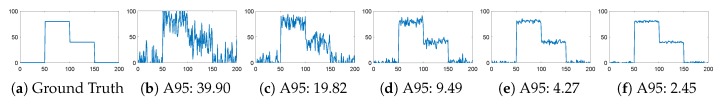
1D use cases for a metric of quantitative comparison, “A〈#N〉”: (**a**) depicts the ground truth signal; (**b**–**f**) show the noise added signals and their A95 errors. The smaller the error, the better the structure is preserved.

For the evaluation, we used a robust accuracy measure as a metric of quantitative comparison, “A〈#N〉” as used in [43]; “A〈#N〉” denotes the depth error at the $N^{\mathrm{th}}$ percentile after the errors are sorted from low to high. We show noisy depth synthetic examples according to different “A〈#N〉” values in Figure 10. For example, if the depth error was 5.0 mm when A95, 95% of the total depth points had errors of less than 5.0 mm. The results of global methods [10,40] had large errors at the dis-occlusion regions, while for the local methods, JBU [9] and ours, we excluded the mask regions that could not compute results with local filters due to large holes or low confidence.

**Figure 11:**
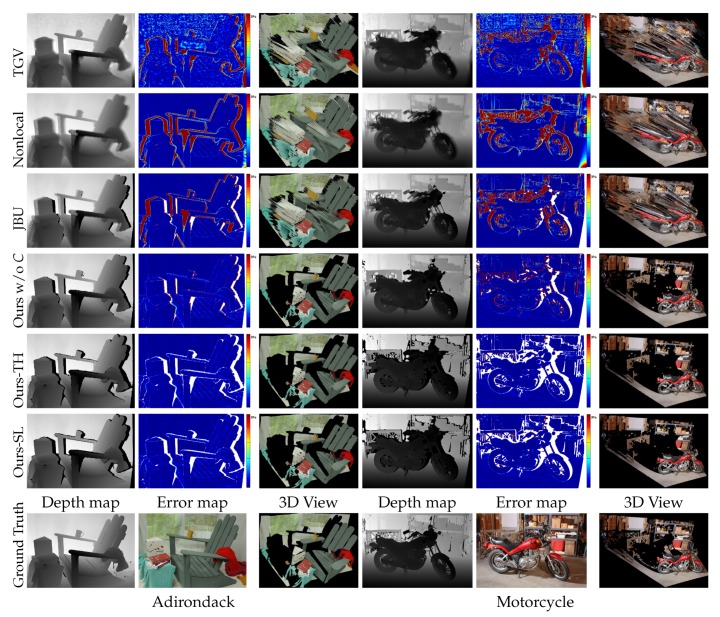
Examples of upsampling results described in Section 3.1. “Ours w/o *C*” denotes our upsampling method without the confidence map. The error maps depict a relative depth error ranging from 0–3% of the maximum depth. The white pixels in the error maps were excluded when the results in Table 1 were computed. We used $\sigma_{s}=20$ pixels, $\sigma_{i}=20$ pixels, and $\sigma_{d}$ =30 mm for the experiment. total generalized variation (TGV) [10], Nonlocal [40], joint bilateral upsampling (JBU) [9], Ours w/o *C* [12], Ours-TH [12].

The major benefit of our approach is a novel depth outlier rejection scheme that gives clear seed depth points. Besides, our scale-aware depth upsampling provided more tolerance on the noisy depth measurements under homogeneous surfaces. Our self-learning framework effectively rejected the remaining ambiguous depth pixels adhering to the boundary region of a large structure without a hand-tuning threshold by a user. The examples of upsampling results and error maps are also shown in Figure 11. Compared to our methods (Ours-TH and Ours-SL), the other methods had a large error and suffered from severe artifacts at the depth boundary regions, which are clearly shown by the 3D view in the figure.

**Figure 12:**
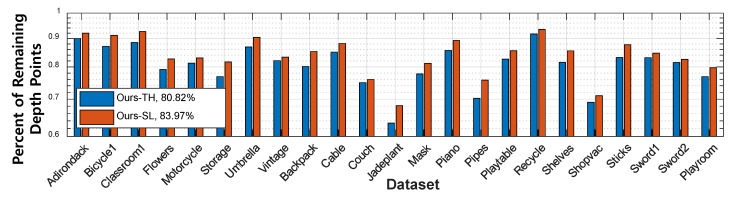
Comparison of valid depth points of Ours-TH and Ours-SL. The percent of remaining depth points after thresholding (blue: Ours-TH, red: Ours-SL).

**Figure 13:**
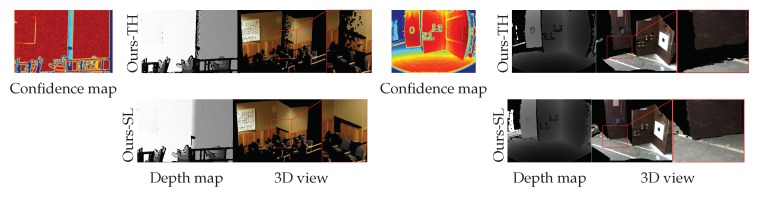
Examples of the qualitative results of the upsampled depth map for comparison between Ours-TH and Ours-SL. The first row depicts the depth maps and 3D point cloud images from Ours-TH [12] and the second row the results of Ours-SL. (**a**) Classroom1 [41]; (**b**) DRC dataset [44].

Table 1 shows the quantitative comparison results. Our methods (Ours-TH and Ours-SL) worked consistently well for both A80 and A95, while the performance of the other methods was significantly degraded for A95. While Ours-TH performed slightly better than Ours-SL in terms of the accuracy metric, as shown in Figure 12 and Figure 13, Ours-TH overly discarded depth points, i.e., it resulted in a low recall performance. In the majority of cases, Ours-TH was simple and worked well with a user-defined threshold value. However, it was infeasible to determine a desirable threshold value for all cases. Furthermore, Ours-TH occasionally discarded too many depth points including well-generated regions. Figure 13a,b shows such cases of Ours-TH. In the 3D views of the figure, we can see that Ours-TH truncated many depth points that were well generated with small errors. The depth map and 3D view of Ours-SL at the bottom of Figure 13a,b retained relatively many depth points with similar errors due to adaptively filtering out the low reliability depth.

### 3.2. Quantitative Evaluation: KITTI

**Figure 14:**
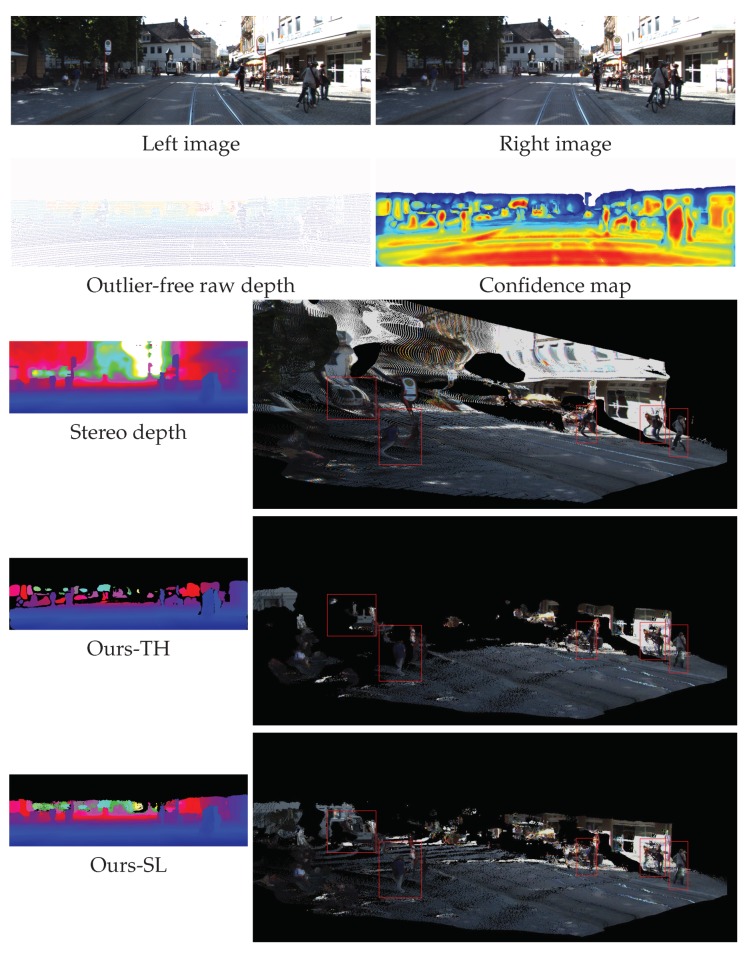
Examples of qualitative results for comparison between Ours-TH and Ours-SL. The two images of the top row show the rectified left and right images, and the images of the second row depict their corresponding LiDAR depth and confidence map, respectively. The images of the third, fourth, and fifth rows present depth maps and their corresponding 3D point cloud. We recommend the readers zoom-in to see the details clearly. Stereo depth [45]; Ours-TH [12].

In this experiment, we show the applicability of our proposed method to improve the accuracy of stereo depth and evaluate the variations of depth accuracy through the KITTI dataset. The initial depth, $D^{0}$, was computed by MC-CNN stereo matching algorithms [45] instead of our upsampling method. Figure 14 shows a qualitative comparison of the depth from Ours-TH and Ours-SL. Even if the depth map of stereo is dense and looks good, it involves large quantities of depth errors at the boundaries of objects and homogeneous texture regions. Compared to the stereo depth in the third row of the figure, Ours-TH preserved the shape of the object structures such as pedestrians and vehicles. However, Ours-TH removed too many reliable depth points that had low confidence values because the single threshold mask did not properly handle the confidence map under various conditions. On the other hand, Ours-SL retained more reliable depth points than Ours-TH while preserving object structures. The depth maps and point cloud images of the fourth and fifth rows show the results of Ours-TH and Ours-SL, respectively.

Table 2 shows the quantitative comparisons. We used different percentages of robust accuracy measure “A〈#N〉” at each dataset to check the stereo depth error at the point of percentage of D1-all. D1-all indicates the median of the percentage of stereo disparity outliers in the overall frames. In terms of D1-all and the robust accuracy, Ours-TH and Ours-SL reduced D1-all and depth errors up to around seven- and four-times, respectively, and Ours-TH had similar or better performance than Ours-SL. However, Ours-TH overly discarded 12∼15% more depth points than Ours-SL. Unlike Middlebury, since the KITTI dataset provides sparse depth information of 3D LiDAR by ground truth, we cannot measure the depth error in all image regions. We can still qualitatively see that many strong depth points were lost, as shown in Figure 13 and Figure 14. We also provide additional results in the supplementary video clip on our web-page: https://sites.google.com/site/iwshimcv/home.

## 4. Conclusions

In this paper, we presented a novel depth upsampling method with the self-learning framework that is designed specifically for filtering out low reliability depth points automatically. In our experiments, we observed that the conventional depth upsampling methods, which do not consider the reliability of upsampled depth points, often produce large depth errors at the object boundaries, which can have an adverse effect on subsequent algorithms that use depth information as input, e.g., depth-based object pose estimation. To deal with this problem, we proposed the self-learning framework, which can automatically predict highly reliable depth points using depth confidence. We showed the effectiveness of our method through two benchmark datasets and also showed that our proposed method can be seamlessly applicable to refine a depth map obtained from stereo matching with favorable performance. Our proposed method has many potential applications by virtue of its robustness, but also has some potential directions that can improve the method further. We leave the discussion as follows:

- Possible applications: According to Qi, C.R. et al. [46], the performance of 3D object detection is highly related to the density of depth. Because our proposed method provides structure-aware dense depth with confidence information, we may expect to improve the performance when applied to 3D object detection.
  Our method will be also helpful for 6D object pose estimation, which is essential to robot-world interaction such as grasping an object. The recent Amazon Picking Challenge [47] showed that a dense depth map and object pose estimation are key components in practical applications. In our previous work [12], we already showed the effectiveness of the high-fidelity depth map on robot-world interaction tasks.
- Discussion and limitations: Filter-based upsampling approaches including our proposed method require some density of measurement points within a local kernel window size to have reliable depth estimation. Thus, depending on the sparsity and the gap among nearest neighbors of seed points, we may need to tune the kernel region-related parameter $\sigma_{s}$ accordingly. Under our sensor configuration, we have shown that our parameter setup is fairly generalizable across many different scenes, but we do not provide other parameter setups for other configurations with different LiDAR models, which may require a different proximity parameter. It would be useful to learn adaptive parameter prediction according to the scene and hardware configuration, which we leave as a future direction.
  Another issue is the computational cost for practical real-world applications. The computation time highly depends on the image resolution and the number of upsampling iterations. In this work, the overall processing time of the proposed method spends about one second to process a 640 × 480 resolution image with five iterations. Some applications that do not require real-time capabilities such as the DARPA Robotics Challenge, https://en.wikipedia.org/wiki/DARPA_Robotics_Challenge, and exploration robots, can utilize our proposed method without significant changes. However, in the case of time-critical applications such as autonomous driving, they may require strict real-time performance. Because most computation is conducted through the greedy convolutional filter operation, it can be parallelized by leveraging modern GPUs.

## Figures and Tables

**Table 1:** Quantitative comparison on Middlebury 2014 dataset [41] with added Gaussian noise using σ = 10 mm. We use $\sigma_{s}$ = 20 pixels, $\sigma_{i}$ = 20 pixels, and $\sigma_{d}$ = 30mm. The best results for each dataset are highlighted in bold, and the second best results are underlined. The unit of value for “A〈#N〉” is millimeter (mm).

Error Metric A80	TGV	Non-Local	Bilat-Eral	Ours-Conf	Ours-TH	Ours-SL	Error Metric A95	TGV	Non-Local	Bilat-Eral	Ours-Conf	Ours-TH	Ours-SL
Adirondack	19.6	9.7	4.7	4.0	**3.3**	3.7	Adirondack	152.3	285.9	160.5	8.4	**7.0**	7.4
Bicycle1	14.5	9.1	4.4	3.6	4.5	**3.3**	Bicycle1	86.8	183.7	116.0	8.0	**6.4**	6.5
Classroom1	40.2	6.3	4.4	3.6	**3.2**	3.4	Classroom1	364.3	99.0	21.0	9.0	**6.3**	7.7
Flowers	64.5	125.5	7.5	3.7	**3.3**	**3.3**	Flowers	1028.0	682.2	575.6	7.6	**5.7**	6.1
Motorcycle	32.0	29.7	7.5	5.7	**5.0**	**5.0**	Motorcycle	388.9	471.8	379.0	15.5	**9.9**	10.6
Storage	44.9	86.1	4.9	3.9	**3.6**	**3.6**	Storage	723.2	1084.8	448.4	10.4	**7.9**	9.0
Umbrella	32.9	8.2	4.6	3.6	**3.5**	**3.5**	Umbrella	259.5	229.4	89.8	7.4	**6.4**	6.8
Vintage	40.3	8.9	**4.3**	4.6	4.4	**4.3**	Vintage	403.8	84.5	17.1	8.1	**7.5**	7.9
Backpack	16.7	7.6	4.5	3.9	**3.5**	**3.5**	Backpack	112.7	126.9	54.3	9.5	**6.0**	6.1
Cable	14.8	6.6	4.3	4.1	**4.0**	**4.0**	Cable	69.5	83.2	57.2	6.9	**6.3**	**6.3**
Couch	119.2	40.3	6.9	5.2	**4.2**	4.5	Couch	820.6	502.6	435.2	15.0	**8.6**	10.2
Jadeplant	96.5	91.8	62.3	6.3	**4.6**	4.9	Jadeplant	540.0	334.0	336.4	96.4	**8.9**	10.5
Mask	34.6	14.8	4.7	4.4	**4.0**	**4.0**	Mask	294.4	251.6	103.8	9.5	**7.0**	7.2
Piano	18.8	8.6	4.7	4.6	4.2	**4.1**	Piano	98.7	94.7	38.3	10.3	8.4	**8.3**
Pipe	156.0	238.3	40.0	8.7	**6.7**	6.9	Pipe	1268.8	1347.2	1194.7	38.2	**12.6**	13.5
Playtable	32.1	24.1	5.8	5.5	**4.6**	4.9	Playtable	340.7	264.8	142.5	12.7	**9.1**	9.8
Recycle	22.6	15.9	5.8	3.6	**3.4**	**3.4**	Recycle	225.2	171.1	153.1	7.4	**6.8**	6.9
Shelves	14.6	6.6	4.1	3.5	3.4	**3.3**	Shelves	75.7	101.1	41.1	7.6	**6.7**	6.8
Shopvac	16.9	8.8	4.5	3.8	**3.3**	3.6	Shopvac	97.2	99.9	40.3	9.7	**6.6**	7.6
Sticks	39.6	6.8	4.1	4.8	**3.5**	4.4	Sticks	120.3	45.8	10.3	10.0	**7.1**	7.3
Sword1	13.3	6.3	4.4	4.2	4.2	**3.7**	Sword1	56.7	26.7	18.9	11.8	**6.8**	7.3
Sword2	22.4	9.4	4.9	4.0	**3.6**	3.8	Sword2	181.1	151.1	106.5	8.3	7.1	**6.2**
Playroom	21.2	8.7	4.6	5.4	**3.7**	4.6	Playroom	170.1	146.0	124.5	13.8	**6.0**	9.3

**Table 2:** Quantitative comparison on the KITTI dataset [42]. We used $\sigma_{s}$ = 20 pixels, $\sigma_{i}$ = 20 pixels, and $\sigma_{d}$ = 0.5 m. pts(%) stands for the percentage of the remaining depth points relative to the total number of stereo depth points in an image. The unit of value for “A〈#N〉” is meters (m).

Dataset	0002				0038				0091				0093			
Error Metric	D1-all	A92	A95	pts(%)	D1-all	A86	A90	pts(%)	D1-all	A87	A90	pts(%)	D1-all	A84	A90	pts(%)
MC-CNN [45]	8.8%	2.27	4.02	100%	13.9%	1.36	2.09	100%	12.6%	1.56	2.24	100%	16.3%	0.85	1.90	100%
Ours-TH [12]	1.4%	0.22	0.32	49.3%	3.8%	0.28	0.47	48.4%	3.4%	0.22	0.35	49.7%	2.5%	0.20	0.32	50.3%
Ours-SL	4.4%	0.29	0.52	61.5%	9.7%	0.44	1.10	63.0%	9.3%	0.34	1.03	64.2%	8.6%	0.26	0.56	62.7%
